# Calibrating Single-Ended Fiber-Optic Raman Spectra Distributed Temperature Sensing Data

**DOI:** 10.3390/s111110859

**Published:** 2011-11-21

**Authors:** Mark B. Hausner, Francisco Suárez, Kenneth E. Glander, Nick van de Giesen, John S. Selker, Scott W. Tyler

**Affiliations:** 1 Department of Geologic Sciences and Engineering, University of Nevada, Reno, MS 172, Reno, NV 89557, USA; E-Mails: suarezf@unr.edu (F.S.); styler@unr.edu (S.W.T.); 2 Department of Evolutionary Anthropology, Duke University, Campus Box 90383, Durham, NC 27708, USA; E-Mail: glander@duke.edu; 3 Civil Engineering and Geosciences, TU Delft, P.O. Box 5048, 2600 GA, Delft, Netherlands; E-Mail: n.c.vandegiesen@tudelft.nl; 4 Department of Biological and Ecological Engineering, Oregon State University, 116 Gilmore Hall, Corvallis, OR 97331, USA; E-Mail: john.selker@oregonstate.edu

**Keywords:** distributed temperature sensing, calibration, hydrology, temperature

## Abstract

Hydrologic research is a very demanding application of fiber-optic distributed temperature sensing (DTS) in terms of precision, accuracy and calibration. The physics behind the most frequently used DTS instruments are considered as they apply to four calibration methods for single-ended DTS installations. The new methods presented are more accurate than the instrument-calibrated data, achieving accuracies on the order of tenths of a degree root mean square error (RMSE) and mean bias. Effects of localized non-uniformities that violate the assumptions of single-ended calibration data are explored and quantified. Experimental design considerations such as selection of integration times or selection of the length of the reference sections are discussed, and the impacts of these considerations on calibrated temperatures are explored in two case studies.

## 1. Introduction

Raman spectra fiber-optic distributed temperature sensing (DTS) technology was originally developed by the oil and gas industries, and has been used since the late 1980s for pipeline monitoring, fire detection and protection, and other industrial applications [1–3]. Since 2006, this technology has been used increasingly to observe environmental temperatures. With the potential for temperature resolutions below 0.1 °C, spatial resolutions of less than 1 m, and sub-minute temporal resolutions [2], commercially available DTS instruments allow researchers to continuously observe spatially distributed temperatures using a single fiber-optic cable as the sensor. Multiple cables in excess of 10 km long can be deployed in the environment and interrogated by a single instrument at spatial and temporal scales that were not feasible before the advent of these systems. In hydrology, DTS systems have been used to observe temperatures in lakes [3], mountain snowpacks [4], groundwater-surface water interactions [5,6], sensible heat fluxes above water surfaces [2,7], soil water content [8,9], tidal estuary behavior [10,11], borehole thermal conductivities [12], and atmospheric processes [13,14].

We will consider the family of DTS instruments that employ Raman spectra scattering of light in an optical fiber. The Raman spectra comprise two frequencies: the Stokes and anti-Stokes, which are each shifted by a predictable wavelength from that of the incident light [2]. The power of Stokes and anti-Stokes backscatter observed at the instrument (hereafter referred to as the “raw data”) can be combined with optical time domain reflectometry (OTDR) principles to estimate temperatures at different positions along the optical fiber [15]. The parameters used to describe the relationships between temperature and the Raman spectra can vary with the operating conditions of the instrument and the optical fiber itself: the instrument’s operating temperature, the quality and consistency of the power supply, the cleanliness and physical condition of any optical connections, and localized strains or bends in the fiber. Many industrial DTS applications (e.g., leak detection and fire protection) need observe only temperature changes which are within the accuracy of standard calibration procedures provided for within the instrument’s standard hardware and software. In the geosciences and environmental sciences, we are faced with the need for much higher accuracy under operating conditions wherein the instrument and optical fiber may be subject to rapid and extreme fluctuations in temperature, humidity, and power supply. While most commercially available DTS instruments include pre-installed calibration routines to translate the Raman signals into temperatures, the internal references on which these routines are based employ loose accuracy under such changing conditions. A DTS that is calibrated to return temperatures with an accuracy of ±0.2 °C under static operating conditions may return temperatures with an accuracy of ±1–2 °C during periods of rapid heating or cooling. In many environmental applications, errors of this magnitude obviate the goals of the installation. In groundwater surface water interactions, for example, a temperature contrast of 10 °C might by typical. To quantify the groundwater influx with an accuracy of ±10% requires temperature observations with a precision of 0.01 °C or better. Hence, robust calibrations are required for the demanding conditions and goals of DTS applied to environmental monitoring.

This paper presents four such algorithms for single-ended DTS data. Single-ended calibration algorithms assume that the calibration parameters remain uniform over the entire length of the fiber. Two data sets collected under very different operating conditions are used to compare the performance of each algorithm by analyzing the accuracy and precision, of the calibrated temperatures. The independent data requirements of each algorithm are listed, and the limitations of the calibration methods are discussed.

## 2. Experimental Design and Calibration Methodologies

**Figure 1.**
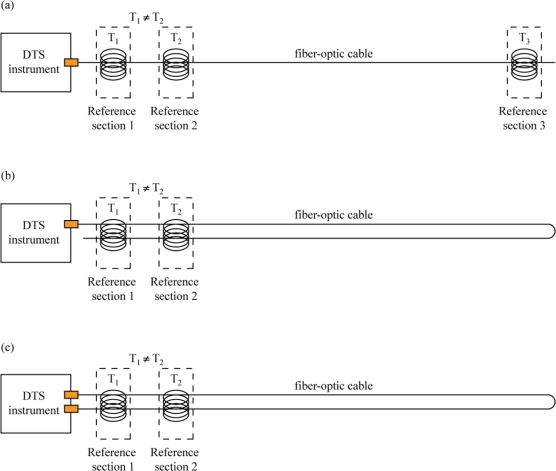
Typical DTS experimental designs. **(a)** Simple single-ended configuration; **(b)** Duplexed single-ended configuration; **(c)** Double-ended configuration.

Fiber-optic cables for DTS studies can be deployed in a number of different configurations, including simple single-ended, duplexed single-ended, or double-ended (Figure 1).

A pure single-ended installation involves a DTS that measures temperatures along a cable strung from the DTS outward, with only one connection to the instrument. A duplexed single-ended installation uses two co-located fibers following the same path which are connected at the end away from the DTS to allow two temperature observations at every point along the cable (one going away from the instrument, and one coming back towards the instrument). In a double-ended installation, the instrument makes observations from both directions on a looped cable that has both ends connected to the DTS. Finally, one may have a double-ended duplexed system in which each end of the duplexed cable is attached to the instrument. In a simple single-ended installation, the calibration routines typically assume uniform light transmission properties along the entire length of the cable, although transient or permanent damage to the fibers, connections between different fibers, and fusion splices can all affect the attenuation of the signal [3]. These localized phenomena, hereafter referred to as “step losses,” are caused when the attenuation of light occurs differently over a short length of fiber than in other places. Step losses can be handled in simple single-ended deployments by calibrating the sections of fiber on either side of the loss separately, but duplexed single-ended and double-ended methodologies both offer the potential to correct for non-uniform attenuation along the cable and are preferred whenever feasible for this reason. The case studies presented here are duplexed single-ended installations.

Commercially available DTS instruments include manufacturer’s calibration routines that rely on reference points or sections of known temperature on the cable. These are typically achieved in the field using sections at known, uniform temperature (e.g., independently logged recirculating water baths) [3]. This length of cable is thus maintained at a uniform temperature that is assumed to be constant on the time scale of the DTS measurement. As we will demonstrate, the calibration must capture effects of attenuation with distance, Raman scattering, and instrument sensitivity. In principle, this would require at least three DTS observations with reference measurements in close proximity, spatially separated along the fiber. The calibration routines provided by the instrument manufacturers typically require two such references adjacent to the instrument, relying on apparent signal attenuation to eliminate the need for a remote observation. This approach is generally insufficient for environmental applications.

DTS calibration reflects the basic physics of backscattering, which we present here. At position *z* (m), the power of the Raman Stokes, *P_S_*(*z*), and anti-Stokes, *P_aS_*(*z*), signals are translated into temperatures according to [16]:

(1) $$T\left(z\right)=\frac{\gamma }{\text{ln}\frac{P_{S}\left(z\right)}{P_{aS}\left(z\right)}+C-\Delta \alpha z}$$

where *γ* (K) represents the shift in energy between a photon at the wavelength of the incident laser and the scattered Raman photon, *C* is a dimensionless calibration parameter that encompasses properties of the incident laser and the DTS instrument itself, and *Δα* (m^−1^) is the differential attenuation between the anti-Stokes and Stokes signals in the fiber. A complete derivation of this equation can be found in the online supplemental material. This paper describes four calibration routines based on Equation (1), as well as the additional information and independent temperature measurements required during the DTS field deployment to use each one.

The calibration procedure seeks to obtain values of the parameters *γ*, *C*, and *Δα* based on the comparison of reported values of *P_S_*(*z*), and *P_aS_*(*z*), and independent temperature measurements at reference locations. With three independent calibration parameters, it is clear that in general three independently measured temperatures are required. The parameter *Δα*, however, may be calculated independently of *C* and *γ*, if one has data from a length of fiber at a uniform temperature (this calculation is derived and described in detail in Section 2.2). With an independently calculated *Δα*, two reference temperatures are sufficient to calculate the remaining two parameters. Typically, the parameters are explicitly calculated based on reference temperature observations. When independent temperature measurements are limited, optimization methods can be used to estimate parameter values by minimizing the differences between observed and calibrated temperatures.

Table 1 describes the four different algorithms that are presented in this paper. The first two return a full set of calibration parameters (*γ*, *C*, and *Δα*), and rely on the explicit calculation of these parameters from three independently measured reference temperatures. The third and fourth algorithms calculate the value of *Δα* independently, and then determine *γ* and *C* simultaneously. These methodologies have varying levels of precision, and are applicable to different installations, depending on the DTS deployment and the independent temperature measurements available. In all cases, however, the calibration methods require *Δα* to be uniform along the optical fiber—any spatial variations in this value must be corrected before calibrating the data. In the case when *Δα* significantly changes along the fiber, it may be more appropriate to use signals retrieved from both directions along the fiber [17], *i.e.*, a double-ended configuration.

### 2.1. Explicit Calculation of Calibration Parameters

The first two algorithms presented below rely on the explicit calculation of three calibration parameters, using three independently monitored reference points (Algorithm #1) or reference sections (Algorithm #2). With three parameters and three known temperatures, it is possible to simultaneously solve a set of linear equations and explicitly calculate the value of each parameter. Using Equation (1) and three points of known distance *z_i_* and temperature *T_i_*, the following system of linear equations can be obtained:

(2) $$\bar{A}\vec{x}=\vec{b}$$

where:

(3) $$\begin{array}{ccc} \bar{A}=\left[\begin{array}{lll} 1 & -T_{1} & T_{1}z_{1}\\ 1 & -T_{2} & T_{2}z_{2}\\ 1 & -T_{3} & T_{3}z_{3} \end{array}\right]; & \vec{x}=\left[\begin{array}{c} \gamma \\ C\\ \Delta \alpha \end{array}\right]; & \vec{b}=\left[\begin{array}{l} T_{1}\;\text{ln}\;\frac{P_{S}(z_{1})}{P_{aS}(z_{1})}\\ T_{2}\;\text{ln}\frac{P_{S}(z_{2})}{P_{aS}(z_{2})}\\ T_{3}\;\text{ln}\frac{P_{S}(z_{3})}{P_{aS}(z_{3})} \end{array}\right] \end{array}$$

If *Ā* is nonsingular, then its inverse can be used to calculate the values of *γ*, *C*, and *Δα*. For the matrix *Ā* to be invertible, it is necessary that *T_1_*, *T_2_*, and *T_3_* include at least two different temperatures. For calibration points that are sufficiently far apart, this condition will be sufficient. Given the matrices *Ā* and *b⃑*, Equation (2) can then be solved for *x⃑* using singular value decomposition.

### 2.2. Independent Calculation of Δα

If three independently measured temperatures are not available, two methods can be used to calculate the value of *Δα* independently of *C* and *γ*. First, the value of *Δα* can be interpolated from a length of fiber at uniform temperature. Because *Δα = α_aS_* − *α_S_*, a relationship between *Δα*, $\frac{P_{S}}{P_{\mathrm{AS}}}$, and *z* can be derived from Beers’ Law. In the absence of other influences, *i.e.*, temperature changes, the ratio $\frac{P_{S}}{P_{\mathrm{AS}}}$ will be attenuated with distance as:

(4) $$\frac{P_{S}(z)}{P_{\text{aS}}(z)}=\frac{P_{0S}\;\text{exp(}-\alpha_{S}\text{z)}}{P_{0\text{aS}}\;\text{exp(}-\alpha_{\text{aS}}z)}$$

where *P_0aS_* and *P_0S_* indicate the power of the anti-Stokes and Stokes signals, respectively, at the point of scatter. Taking the natural logarithm of Equation (4) yields:

(5) $$\text{ln}\frac{P_{S}}{P_{\mathrm{AS}}}\left(z_{2}\right)=\text{ln}\frac{P_{S}}{P_{\mathrm{AS}}}\left(z_{1}\right)+\Delta \alpha \left(z_{2}-z_{1}\right)$$

Equation (5) relates the ratio $\frac{P_{S}}{P_{\mathrm{AS}}}$ to distance along the fiber. The value of *Δα* can then be calculated as the slope of a line fitting the observed values of $\text{ln}\left[\frac{P_{S}}{P_{\mathrm{AS}}}(z)\right]$ as a linear function of distance *z* for a section of fiber at uniform temperature. In DTS applications, the accuracy of this calculation will depend primarily on the uniformity of the temperature and the linearity of the Raman spectra data, but it is also related to the number of data points considered in the regression (these impacts are discussed in Section 4.2).

In the absence of an extensive reference section of fiber, *Δα* can be calculated from two separate points (distances *z_1_* and *z_2_*) at the same temperature by rearranging Equation (5) to yield:

(6) $$\Delta \alpha =\frac{\text{ln}\frac{P_{S}\left(z_{1}\right)}{P_{aS}\left(z_{1}\right)}-\text{ln}\frac{P_{S}\left(z_{2}\right)}{P_{aS}\left(z_{2}\right)}}{z_{2}-z_{1}}$$

In Equation (6), the parameters *γ* and *C* are eliminated from the equation, and the value of *Δα* can be calculated from the Raman spectra data. The accuracy of the calculated *Δα* value will be directly proportional to the distance between the two reference points—the farther apart the two observations are, the more representative *Δα* is of the fiber between the points.

Once the value of *Δα* has been calculated, the values of *γ* and *C* are determined. When two reference points with different temperatures are available, these parameters can be calculated explicitly by simultaneously solving Equation (1) at each reference point [16]. If two different reference temperatures are not available (or the two reference points are at similar temperatures), the parameters must be determined through optimization. In this case, we elect to minimize the objective function:

(7) $$\mathrm{Obj}=\mathrm{MB}=\frac{1}{\mathrm{mn}}\left|\sum_{1}^{m}\sum_{1}^{n}\left(T_{\mathrm{DTS}}-T_{\mathrm{obs}}\right)\right|$$

where *MB* is the absolute mean bias of the calibrated data, *n* is the number of independent temperature observations, *T_DTS_* is the calibrated DTS temperature at each observation point and *T_obs_* is the independently measured temperature at the same point. This objective function is the *MB* of *(mn)* temperature measurements in *m* baths containing *n* observations each.

### 2.3. Reference Points *vs.* Reference Sections

In the previous discussion, we have frequently referred to “reference points” along the cable. While single points do provide a basis for calibrating DTS data, they are not ideal, especially for optimization algorithms. For more precise calibration parameters and reduced overall error, the calculations above can all be made using sections of fiber at uniform temperature rather than individual points. For each reference section, representative values for distance (z*) and log power ratio ( $\text{ln}\left[\frac{P_{S}*}{P_{\mathrm{AS}}}\right]$) must be determined. As the inverse of temperature varies linearly with both *z* and $\text{ln}\left[\frac{P_{S}}{P_{\mathrm{AS}}}\right]$ (Equation (1)), the representative values z* and $\text{ln}\left[\frac{P_{S}*}{P_{\mathrm{AS}}}\right]$ are best calculated as the arithmetic mean of each of these parameters along the reference section:

(8) $$z*=\frac{1}{n}\sum_{i=1}^{n}z_{i}$$

(9) $$\text{ln}\frac{P_{S}*}{P_{aS}}=\frac{1}{n}\sum_{i=1}^{n}\text{ln}\frac{P_{S}(z_{i})}{P_{aS}(z_{i})}$$

Using reference sections rather than single points can greatly improve both the precision and accuracy of the recalibrated data [16]. Tyler *et al.* [3] recommend reference sections of at least ten times the sampling interval for calibration purposes. The effects of reference section length on calibrated temperature resolution are discussed in Section 4.2.

### 2.4. Evaluating Calibrated Data

Once a data set has been processed, it is important to establish both the accuracy and the precision of the calibrated data. Independent temperature observations are required to perform this evaluation, and the accuracy and precision of these independent observations will limit the accuracy and precision of the user-calibrated data. In the example data sets presented below, we use *MB* (described above), the root mean square error (*RMSE*), and the duplexing error (*E_dup_*) as metrics for the quality of the calibration. The *RMSE* of *n* temperature observations is calculated as:

(10) $$\mathrm{RMSE}=\frac{1}{n}\sqrt{\sum_{i=1}^{n}{\left(T_{i}-T\right)}^{2}}$$

where *T* is the known temperature and *T_i_* is the DTS-estimated temperature at a given point.

For deployments like the duplexed installations presented here, two temperature observations are made at every point along the cable and *E_dup_* can also be used as a metric of calibration. Duplexing error is calculated according to

(11) $$E_{\mathrm{dup}}=\frac{1}{n}\left|\sum_{1}^{n}T_{n1}-T_{n2}\right|$$

in which *n* is the total number of observations along the entire fiber-optic cable and *T_n1_* and *T_n2_* are the two temperature observations at the same point. Because the duplexing is not perfect, areas with sharp spatial temperature gradients (e.g., where cable enters or leaves calibration baths) may have to be omitted from this equation. When these areas are omitted, *E_dup_* can be calculated along the entire cable.

Under ideal conditions, at least four reference sections of known temperature are needed to evaluate the quality of the user-calibrated data: three for calibration (making the explicit calculations possible) and a fourth for validation. These sections do not have to have four different temperatures; two different temperatures suffice, so a cable may loop back through the same warm and cold baths it went through at its beginning. The *RMSE* observed in the calibration sections provides an assessment of the accuracy of the instrument noise, and the *MB* observed in a carefully selected validation section can reveal any systematic inaccuracies in the calibrated data. Validation sections should be selected with the intent of finding the most unreliable sections of the calibrated fiber. Because the calibration is sensitive to distance and the temperatures in the baths are constrained, the validation sections for a single-ended calibration should be as far away from the calibration baths as possible. *RMSE*, *MB*, and *E_dup_* are expressed in the same units as the DTS observations, making them intuitively understandable metrics for calibration.

Every DTS calibration method, whether presented in this paper or included in the instrument’s software, depends on two or more reference points on the fiber. Because these methods are dependent on not only the reference points, but also on the uniformity of the fiber between these points, it is prudent to assume that a valid calibration is obtained only on the section of fiber falling between the first and last reference point.

### 2.5. Static and Dynamic Calibrations

On many commercially available DTS instruments, it is possible to define either a static or a dynamic calibration. A static calibration is based on a single set of parameters generated from data taken over some initial integration period. The static calibration assumes that the parameters do not change with time, and applies the parameters derived from the initial trace to each time slice of the DTS data. While this may be suitable for a DTS in a carefully controlled environment, or for an application requiring limited accuracy (e.g., fire detection), a static calibration is not likely to return consistently reliable data in environmental applications. Static calibrations are subject to drift, especially in settings where operating conditions change cyclically over time (e.g., diurnal temperature swings). All the components of DTS instruments (e.g., power-supplies, laser, detectors, amplifiers) are temperature sensitive, and these sensitivities are corrected internally by continually comparing the temperature of an internal reference coil (typically 50–100 m in length) to a precision thermometer. When temperatures change quickly, the true mean temperature of the reference coil can diverge from that of the monitoring thermometer, leading to erroneous offsets of the reported temperature. If accuracy is critical, it is essential to eliminate these errors through dynamic calibration. We note that any mismatch between the true internal reference coil temperature and its monitoring thermometer will result in an erroneous offset in the DTS reported temperatures.

While a static calibration assumes that the parameters are constant in time, a dynamic calibration recalculates the three parameters during each timestep of the data acquisition. Dynamic calibrations rely on reference sections defined by the user while the instrument is being launched; instruments may use constant-temperature ice baths or integrated temperature probes to create fully dynamic calibrations [3].

### 2.6. Correcting for Step Losses

Step losses in the raw Raman spectra data can significantly impact calibration, introducing artificial offsets and slopes into the calibrated temperatures. The case studies presented here each include at least one step loss, which must be corrected prior to calibration. Step losses in the case studies presented here were removed by applying a calculated linear offset to the quantity $\text{ln}\left[\frac{P_{S}}{P_{\mathrm{AS}}}\right]$ at each point. This offset is calculated by matching the power ratio at two points immediately before and after the localized step loss; given that these two points are at the same temperature and separated by less than ten meters (differential attenuation is typically on the order of 10^−5^ m^−1^), the power ratio at these two points should be nearly identical. For each timestep, the offset sufficient to match the farther of these points to the closer one was applied to every point from the end of the localized step loss to the end of the cable. Calibration algorithms described below were then applied to the adjusted power ratios.

### 2.7. Calibration Algorithms

In each of the case studies presented, instrument-calibrated DTS data were compared to independent temperature observations and user-calibrated data. Each case study included four calibration baths: two warmer and two cooler baths. Four dynamic calibration methodologies were compared to a single static user calibration. The first two calibration methodologies were explicit calculations based on Equation (1). Algorithm #1 used three points as the basis for the calibration, and Algorithm #2 used three reference sections of 16–21 meters each. In both of these methodologies, the two cooler baths and the near-instrument warmer bath were used to calibrate temperatures, and the far warmer bath was used as the basis for the validation. In Algorithm #3, the value of *Δα* was interpolated from the near cool and far warm baths according to Equation (5), and the values of *γ* and *C* were then explicitly calculated using the same two baths as the calibration references. These data were validated using the 32–42 observation in the near heated and far ambient baths (16–21 observations each). Algorithm #4 calculated the value of *Δα* from the two reference sections in the cooler baths using Equation (6), and used the MATLAB function *fminsearch* [18]*,* which uses a simplex method [19] to determine the values of *γ* and *C* that minimize the absolute mean bias (Equation (7)) of the defined calibration sections [19]. Calibration constants for the static calibration were calculated as the mean of the set of parameters generated by Algorithm #2.

For each of these four calibration algorithms, the *RMSE* and *MB* in the calibration baths and the validation baths were calculated separately for each time step. The mean, standard deviation, and range of these metrics over the entire 48 hour deployment are reported for each method, as well as for the instrument-calibration. The *RMSE* and *MB* of the static calibration were calculated for each time step, and temporal changes in these were compared to the operating conditions in each installation.

## 3. Case Studies and Sample Calibrated Data Sets

The calibration algorithms presented in this paper have been applied to two different DTS data sets: a laboratory installation monitoring a salt-gradient solar pond at the University of Nevada, Reno, and a field deployment measuring temperatures in the dry forest canopy of a research center located on *Hacienda La Pacifica*, a privately owned ranch in Costa Rica. The two data sets include very different fiber characteristics and resolution, and the contrast between the two serves to illustrate the range of results that may be expected from these methods.

### 3.1. Laboratory Data from a Salt-Gradient Solar Pond

An experimental salt-gradient solar pond was constructed to investigate sustainable water production using solar energy. To have controlled conditions, the solar pond was built inside a laboratory and was subject to high-density discharge lamps that were turned on for 12 hours per day [20]. The pond was instrumented with a variety of sensors to monitor its performance, including a vertical high-resolution DTS system [16]. This laboratory setup provided nearly ideal conditions for a DTS installation, with four calibration baths in a well-controlled environment.

Data were collected in the solar pond with a Sensornet Sentinel DTS instrument (Sensornet LTD., Hertfordshire, UK) featuring an external multiplexer. The Sentinel is equipped with two reference thermometers constructed from 100 Ω platinum PT100 sensors (Sensornet LTD, Hertfordshire, UK), and has a manufacturer’s specified operating range of 0–40 °C. The reference thermometers have an accuracy of 0.1 °C and a precision of 0.02 °C, and were previously calibrated against a NIST traceable thermometer (±0.05 °C; Control Company, Friendswood, TX, USA). The PT100s were placed in two calibration baths to provide the independently measured temperatures required for calibration. Temperature observations were made on a 1 meter sampling interval with a 300 s integration time.

**Figure 2.**
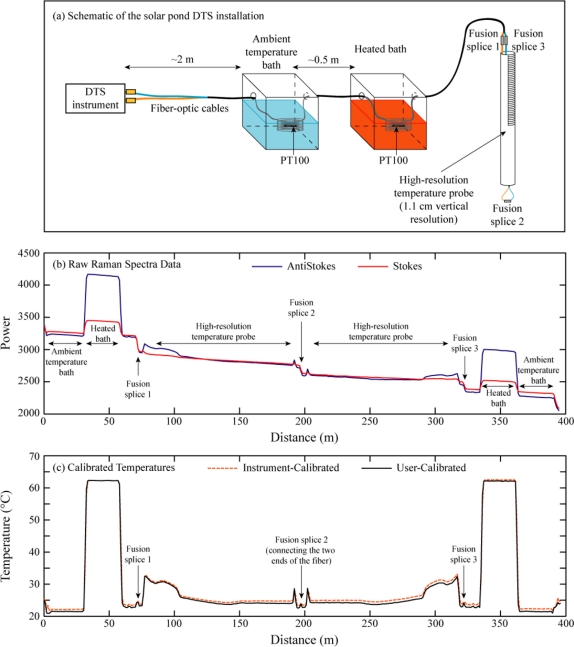
The laboratory solar pond installation. **(a)** Schematic of the laboratory configuration; **(b)** Raw Raman spectra data (recorded by the DTS instrument in arbitrary units linearly related to the power of the scattered signals) and the locations of calibration baths for a sample 5 minute DTS trace; **(c)** Temperatures (both instrument-calibrated and user-calibrated) and the locations of fusion splices for a sample 5 minute DTS trace.

In the solar pond installation, the duplexed (two fibers co-located in a single cable), loose-tube fiber-optic cable (AFL Telecommunications, Duncan, SC, USA) passed through a recirculated water bath at ambient temperature and then through a microprocessor-controlled elevated-temperature water bath (Precision 280, ThermoElectric Corporation, Waltham, MA, USA). The cable then passed through a protective conduit into a junction box above the pond. Fusion splices connected the two fibers to a high-resolution temperature probe constructed with the same cable. In this probe, the cable was wrapped tightly around a 2-inch polyvinyl chloride (PVC) pipe; the spiral wrapping allowed the instrument’s 1 meter spatial integration limit to return temperature observations with a vertical resolution of 1.1 cm. The high-resolution probe extended from approximately 20 cm above the surface of the 1 meter deep pond to the bottom; the fiber-optics returned to the junction box up the inside of the PVC pipe. In the junction box, the two ends of the fibers in the cable were spliced together to form a single, long fiber. Thus, the fiber path was from the instrument, through the ambient temperature bath, to the heated bath, to the junction box, from the surface of the pond to the bottom, and back to the junction box; after passing through the fusion splice in the junction box, the second half of the duplexed trace is the mirror image of the first (Figure 2(c)).

The greater sensitivity of the anti-Stokes signal to temperature is readily apparent from the data before and after the fiber entered the heated baths (Figure 2(b)). Forty-eight hours of continuous temperature data were collected using 5 minute integration times. The dynamic instrument calibration was based on matching the temperatures of the reference sections in the ambient temperature bath to the temperatures recorded by the integrated PT100 in that bath. The temperature of the heated bath was used to validate the instrument calibration. We recalibrated the raw data returned by each of the 575 traces collected during the 48 hour experiment using the four calibration algorithms (see Table 1) and compared the user-calibrated data to the instrument-calibrated temperatures and the independent temperature observations.

### 3.2. Field Data from an Ecological Research Center

To investigate thermoregulation of howling monkeys (*Alouatta palliata*), a field expedition was carried out to monitor vertical temperature variations in the canopy of their forest habitat (located on *Hacienda La Pacífica*, in Costa Rica). In this field installation, a fiber-optic cable passed over the branches of a tree at an approximate elevation of 15 m and then returned back to ground level.

This application used a Sensornet Oryx DTS instrument (Sensornet LTD., Hertfordshire, UK) with an operating temperature range between −20 and 60 °C. This instrument is similar to the Sensornet Sentinel DTS instrument, but better able to withstand the temperatures and power limitations inherent to field work. The Oryx includes PT100 sensors identical to those employed with the Sentinel, and these were again used to measure temperatures in two reference baths. The instrument was housed in a protected hard case with a computer that continuously downloaded and archived the collected data. Temperatures were observed with a 1 m sampling interval and 30 s integration time.

A 6 mm OD armored FO PBT patchcord 50/125 duplexed cable (Kaiphone Technology Co., LTD., Taipei, Taiwan) was used to provide reliable performance under the field conditions. The fiber and central capillary tube are the same as those used in the solar pond experiment, but the core element was wrapped with a spiral steel shield to protect the fibers from impingements, aramid strands around this to protect the fibers from tension, and an HDPE outer sheath to protect the entire assembly from abrasion. The cable left the instrument, passed through a recirculated cold water bath, an unmonitored ambient temperature bath, up into the forest canopy and back down, through the unmonitored bath a second time, and into a second circulated, monitored ambient temperature bath. On the other side of this bath, a fusion splice joined the two fibers together, resulting in a mirror image data set similar to that seen in the solar pond experiment.

**Figure 3.**
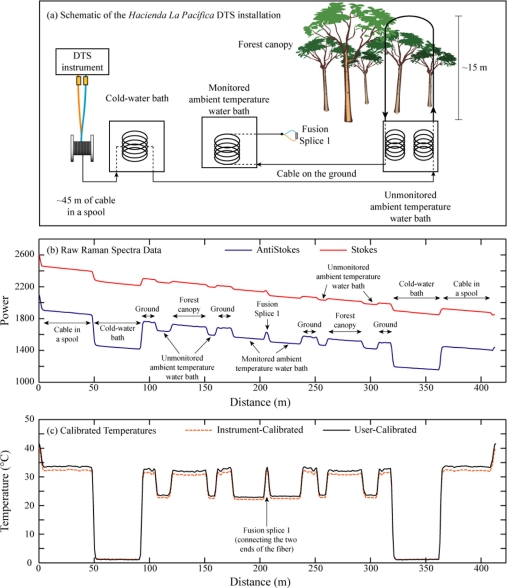
*Hacienda la Pacifica* DTS installation. Note that in addition to the monitored calibration baths, the cable makes multiple passes through an unmonitored temperature bath at approximately 110, 155, 255, and 300 m. **(a)** Schematic of the field installation; **(b)** Raw Raman spectra data (recorded by the DTS instrument in arbitrary units linearly related to the power of the scattered signals) and the locations of calibration baths for a sample 30 second DTS trace; **(c)** Temperatures (both instrument-calibrated and user-calibrated) and the locations of fusion splices for a sample 30 second DTS trace.

Figure 3 shows the raw data from a single trace from the *Hacienda la Pacifica* installation, as well as the instrument-calibrated temperatures from the same trace. The locations noted above are marked on the figure. Forty eight hours of data were collected at this location. To minimize power consumption in the field, the data were collected using an integration time of 30 seconds every four minutes. The data set presented here therefore consists of 720 individual traces, each with a 30 second integration time.

## 4. Results and Discussion

The calibration metrics from the laboratory and the field installations are shown in Tables 2 and 3, respectively. *RMSE* and *MB* for both the validation and calibration baths are reported as the mean ± the standard deviation of the 575 (laboratory) or 720 (field) traces. The duplexing error is not specific to the calibration or validation baths, and is reported once for each calibration algorithm as the mean ± the standard deviation of the total number of traces.

### 4.1. Calibration Metrics and Discussion

The data in Tables 2 and 3 clearly indicate that the explicit calculation of calibration parameters consistently returns the most accurate and least biased temperature measurements in both installations. However, this method also presents the greatest challenge for the researcher—even assuming multiple passes of the cable through the calibration baths, at least two calibration baths must be maintained and constantly monitored at distinctly different temperatures. For long-term installations (longer than approximately 24–36 hours, depending on the on-site weather conditions) this requires either heating (consuming additional power) or cooling (periodic replenishment of ice or power-driven refrigeration) of at least one bath. While this is a simple task in the laboratory, it can be challenging for field installations.

The need for step loss corrections is apparent from an examination of the duplexing error (Equation (11)) in Tables 2 and 3. The value of *Δα* can be strongly influenced by the physical condition of the fiber itself, and it can also change significantly over the length of the deployed cable. If the cable is damaged, subjected to localized strains, or bent around a too-sharp radius, the signal losses at this point will be greater than the signal losses over the remainder of the cable and the assumption of uniform attenuation is violated by the damaged area. Fusion splices, for example, may introduce small (e.g., in the laboratory experiment at 72, 195, and 318 m) or large (e.g., meter 204 in the field experiment) losses. These issues must be attended to prior to undertaking calibration.

In these case studies, the step losses were corrected as described above in Section 2.6. Such a correction is best checked by examining the duplexing error; in a non-duplexed cable, the correction can be checked by confirming that reported temperatures immediately before and after the junction are identical. In the field data presented here, the single step loss was located at the point where the two duplexed fibers were fused together, and the assumption of identical temperature on either side of the step was valid. In the laboratory, only one of the three steps was found at the far end of the cable where the two fibers were spliced together. Because the other two steps were not as well controlled, the loss correction routine used for the laboratory data introduced a greater uncertainty than the routine used for the field data. This uncertainty is reflected in the duplexing error, which is nearly always greater in the laboratory than in the field. Despite the additional uncertainty, the step corrections applied to the laboratory data still resulted in improvements above the manufacturer’s calibration—it is a necessary step to ensure usable data.

The step correction is feasible with fully duplexed installations, and may be possible for pure single-ended installations in which the entire cable is accessible. It is not always feasible, however, to maintain multiple independent temperature loggers throughout the period of deployment. Temperature profiling in boreholes or streambeds, for example, often cannot be addressed in this manner. When field conditions prevent the use of multiple calibrations, double-ended DTS configurations may be used to mitigate the effects of non-uniform attenuation [3]. In a fiber with a non-uniform differential attenuation, the term *Δαz* in Equation (1) should be expressed as the definite integral $\int_{0}^{z}\Delta \alpha (u)\mathrm{du}$ [17]. Double-ended DTS observations, which measure the anti-Stokes and Stokes power in both directions through a given length *Δz* of fiber, make it possible to calculate the spatially integrated differential attenuation for each spatial sampling interval individually [17], and such routines can better account for localized fiber anomalies [2]. Although it is outside the scope of this paper, there is a real need for double-ended calibration routines that can be easily used when multiple single-ended calibrations are not feasible; these issues will be addressed in a forthcoming article.

For applications that do not require the accuracy offered by the three-section explicit calibration (or for installations in which three reference sections are not available), the independent measurement of *Δα* as illustrated by calibration algorithms #3 and #4 offers an alternative, but suffers a significant loss of accuracy. The reliability of the recalibrated temperatures should therefore be considered carefully. Under some conditions, these methods may improve the *RMSE* or *MB* when compared to the manufacturer’s calibration (e.g., algorithm #3 in the field); in other settings, the duplexing error may be reduced at the cost of greater *RMSE*. The utility of these data must ultimately be determined by the user. Without additional reference temperatures (e.g., validation baths, occasional points of known temperature, known points of identical temperature), it is difficult to understand or to quantify the accuracy of the recalibrated temperatures.

### 4.2. Length of Fiber in Reference Sections

**Figure 4.**
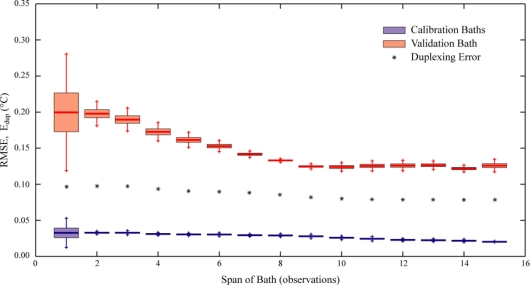
Calibration variations due to length of reference sections. The box and whisker plots indicate the mean *RMSE* (the heavy horizontal line), three standard deviations (the shaded box), and five standard deviations (the whiskers) in both the calibration baths (blue) and the validation baths (red). The duplexing error in the validation baths is indicated by the black asterisks.

Tyler *et al.* [3] recommend the use of at least ten observations in the reference sections used for calibration. Conceptually, it is desirable to avoid adding noise from the measurements in the calibration bath to all the rest of the data. Since the noise drops with the square-root of the length of the measurement section, employing ten observations will reduce the noise added by the baths to less than one third of the noise of each reading to which these calibration data are applied. The validity of this recommendation is illustrated in Figure 4, which shows the metrics for user-calibrated data from the solar pond installation based on reference sections consisting of 1 to 15 observations. For reference sections of fewer than ten points, it is clear that using a longer reference section improves the *RMSE* of the validation bath in the user-calibrated data. In addition to reducing the mean *RMSE*, the longer reference sections also yield less scattered results, as indicated by the consistently tightening range of *RMSE* values, and the duplexing error improves slightly with the number of references. At the same time, the variability of the calibration baths is also somewhat reduced, although this variability is significantly less than that of the validation baths. When the reference sections reach ten observations, however, the validation *RMSE*s gain little benefit from using longer sections as the basis for calibration. At this range, the mean *RMSE* remains fairly constant at approximately 0.12 °C and the duplexing error appears to stabilize at approximately 0.08 °C. It is instructive to look at the calibration *RMSE* in this range of section length as well; the calibration *RMSE* continues to improve with longer reference sections, but only slightly—from less than 0.03 °C to approximately 0.02 °C.

The use of reference sections rather than points is intended to minimize the uncertainty of the temperatures on which the calibration is based. It is important to note that the quantitative aspects of this analysis are applicable only to this particular combination of instrument, fiber, calibration baths, and temperature loggers. Although we expect other DTS deployments to exhibit similar behavior, the distances over which the shift occurs will always be dependent on the instruments and materials used.

### 4.3. Static and Dynamic Calibrations

Table 4 shows the mean *RMSE*, temporal repeatability, and spatial repeatability of both the laboratory and the field data based on a static calibration. In both cases, the values of the parameters *Δα*, *γ*, and *C* were taken as the mean of the values derived from the three-section explicit calculation method used above (algorithm #2). The static calibration metrics are clearly worse than the results of the dynamic calibrations presented in Tables 2 and 3, but the difference between the laboratory and field results is again striking. In the laboratory, the *RMSE* values for the calibration and validation baths rose by factors of 3 and 2, respectively, when a static calibration was applied. In the field data, these errors increased by factors of 10 and 3, respectively.

**Figure 5.**
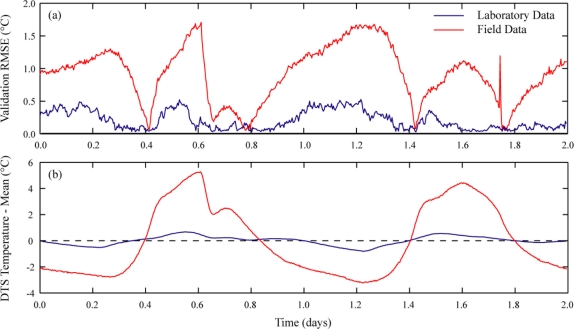
The influence of instrument temperatures on static calibrations. **(a)** Validation bath *RMSE* of the static calibrations for the two case studies presented here. **(b)** Difference between the mean instrument temperature and the instrument temperature during the integration time.

Dynamic calibrations take into account the changes in operating conditions experienced by the DTS instrument. Figure 5 shows the deviation from the mean of the internal temperatures of the DTS instruments (as recorded by the instruments themselves) over each 48 hour deployment, along with the time series of *RMSE* of the static calibration over the same time period. The laboratory instrument, which showed a relatively small increase in error with a static calibration, was maintained at a fairly constant temperature—the instrument temperature was always within a range of less than 1.5 °C. The field instrument, however, was subjected to a diurnal temperature swing of up to 8.5 °C, and this temperature variation is reflected in the quality of the recalibrated temperatures. While dynamic calibrations can improve data collected under controlled conditions, they are critical for field applications or other settings where the operating conditions can change significantly.

## 5. Conclusions

With the growing popularity of DTS instrumentation in hydrologic research, there is a definite need for post-processing calibration routines that can return more precise temperature observations than the manufacturer’s calibrations performed with the instruments. The derivations and algorithms presented in this paper are intended to begin filling that void. When used with raw DTS data collected from single-ended deployments with suitable independent temperature observations, the methods presented here can refine temperature resolution significantly beyond the resolution obtained from the instrument-calibration. The choice of calibration method depends on the temperature resolution desired, as well as the availability of suitable independent observations for use as reference temperatures.

While the methods presented here are capable of improving the temperatures derived from many field campaigns, they are not appropriate for all deployments. In particular, simple single-ended field deployments with non-uniform losses in the cable cannot be easily addressed without additional information. Duplexed single-ended experiments can provide a more robust dataset, but multiple step losses may make correction difficult even on these installations. Double-ended measurements can offer a better opportunity to refine the data, minimizing the impact of multiple step losses on the resulting temperatures, and there is a significant need for double-ended calibration routines that can better handle these kinds of errors.

Finally, the quality of the temperatures returned by any calibration method will depend on the quality of the DTS experimental design, the DTS instrument itself, the field deployment, and independent temperature measurements. The examples presented in this paper represent two very different conditions, but similar problems appear in each case study. It is our hope that these studies provide some lessons about how to design successful DTS experiments.

## Supplementary Information



## Figures and Tables

**Table 1.** Brief description of the four calibration algorithms presented in this work.

Algorithm Number	Calibration Methodology
1	Explicit calculation of parameters from a set of three reference points
2	Explicit calculation of parameters from a set of three reference sections
3	Independent calculation of Δα by interpolation in two reference baths, with explicit calculation of γ and C from those two baths
4	Independent calculation of Δα from matched reference baths, with optimization of γ and C from those two baths

**Table 2.** Calibration Metrics for the Salt-Gradient Solar Pond (n = 575, 1 m spatial sampling interval, 300 s integration time, 16 points in each reference and validation section). **(a)** Calibration metrics recorded in the calibration baths. **(b)** Calibration metrics for the validation baths. While the manufacturer’s calibration did not include the step loss correction, the 4 sample calibration algorithms were run on the corrected data.

**(a) Calibration Metrics**			

Calibration Algorithm	RMSE (°C) μ ± σ (range)	Bias (°C) μ ± σ (range)	

Manufacturer’s Calibration	0.387 ± 0.012 (0.354 to 0.430)	0.279 ± 0.015 (0.244 to 0.323)	
1	(Calculated explicitly: no variation in calibration baths)		
2	0.041 ± 0.007 (0.025 to 0.065)	<10^−4^	
3	0.036 ± 0.006 (0.021 to 0.063)	<10^−4^	
4	0.184 ± 0.128 (0.035 to 0.510)	−0.155 ± 0.156 (−0.509 to 0.153)	

**(b) Validation Metrics**			

Calibration Algorithm	RMSE (°C) μ ± σ (range)	Bias (°C) μ ± σ (range)	Duplexing Error (°C) μ ± σ (range)

Manufacturer’s Calibration	0.793 ± 0.034 (0.721 to 0.909)	0.792 ± 0.034 (0.721 to 0.909)	0.1622 ± 0.024 (0.094 to 0.231)
1	0.131 ± 0.066 (0.034 to 0.374)	−0.011 ± 0.139 (−0.371 to 0.293)	0.099 ± 0.023 (0.043 to 0.169)
2	0.131 ± 0.052 (0.035 to 0.271)	0.016 ± 0.132 (−0.267 to 0.237)	0.087 ± 0.016 (0.042 to 0.131)
3	1.22 ± 0.550 (0.071 to 2.82)	−0.019 ± 0.031 (−0.100 to 0.071)	0.818 ± 0.364 (0.061 to 1.87)
4	1.99 ± 0.247 (1.43 to 2.59)	−1.99 ± 0.247 (−2.59 to −1.43)	0.074 ± 0.014 (0.042 to 0.116)

**Table 3.** Calibration metrics for the field deployment at *Hacienda la Pacifica* (n = 720, 1 m spatial sampling interval, 30 second integration times, 21 points in each reference and validation section). **(a)** Calibration metrics for the three calibration baths. **(b)** Calibration metrics for the validation bath. The sample algorithms were run on step-corrected data, while the manufacturer’s calibration reflects only the uncorrected data.

**(a) Calibration Metrics**			

Calibration Algorithm	RMSE (°C) μ ± σ (range)	Bias (°C) μ ± σ (range)	

Manufacturer’s Calibration	0.555 ± 0.064 (0.446 to 0.876)	−0.329 ± 0.143 (−0.858 to −0.058)	
1	(Calculated explicitly: no variation in calibration baths)		
2	0.064 ± 0.010 (0.046 to 0.207)	<10^−4^	
3	0.060 ± 0.008 (0.044 to 0.148)	<10^−4^	
4	0.058 ± 0.009 (0.042 to 0.191)	<10^−4^	

**(b) Validation Metrics**			

Calibration Algorithm	RMSE (°C) μ ± σ (range)	Bias (°C) μ ± σ (range)	Duplexing Error (°C) μ ± σ (range)

Manufacturer’s Calibration	0.583 ± 0.070 (0.345 to 0.804)	−0.580 ± 0.070 (−0.801 to −0.338)	0.136 ± 0.008 (0.110 to 0.159)
1	0.120 ± 0.060 (0.039 to 0.420)	0.040 ± 0.112 (−0.286 to 0.413)	0.081 ± 0.061 (0.000 to 0.298)
2	0.108 ± 0.048 (0.038 to 0.324)	0.043 ± 0.091 (−0.223 to 0.318)	0.065 ± 0.021 (0.000 to 0.118)
3	0.386 ± 0.221 (0.052 to 1.21)	0.209 ± 0.182 (−0.403 to 0.733)	0.491 ± 0.324 (0.002 to 1.66)
4	2.65 ± 0.429 (1.50 to 3.26)	−2.65 ± 0.429 (−3.26 to −1.50)	0.109 ± 0.020 (0.038 to 0.171)

**Table 4.** Calibration metrics for static calibration.

	Laboratory Data		Field Data	
Metric	Calibration	Validation	Calibration	Validation
RMSE (°C) μ ± σ (range)	0.146 ± 0.086 (0.031 to 0.359)	0.230 ± 0.127 (0.033 to 0.635)	0.747 ± 0.429 (0.038 to 2.29)	0.796 ± 0.463 (0.048 to 2.19)
MB (°C) μ ± σ (range)	−0.001 ± 0.159 (−0.320 to 0.355)	−0.165 ± 0.200 (−0.634 to 0.411)	−0.003 ± 0.855 (−2.20 to 1.23)	0.370 ± 0.941 (−1.71 to 1.68)
Duplex. Error (°C) μ ± σ (range)	0.102 ± 0.076 (0.042 to 0.258)		0.134 ± 0.042 (0.070 to 0.399)	
