# Supplementary material for: Calibrating Single-Ended Fiber-Optic Raman Spectra Distributed Temperature Sensing Data
Source: Sensors (Basel). 2011 Nov 21;11(11):10859–79. doi: 10.3390/s111110859 (PMC3274318; doi:10.3390/s111110859)
Supplement: Supplementary file 1 [file sensors-11-10859-s001.pdf]

Supplementary Information

Calibrating Single-Ended Fiber-Optic Raman Spectra Distributed Temperature Sensing Data

Mark B. Hausner [1,*], Francisco Suárez [1], Kenneth E. Glander [2], Nick van de Giesen [3], John S. Selker [4] and Scott W. Tyler [1]

[1] Department of Geologic Sciences and Engineering, University of Nevada, Reno, MS 172, Reno, NV 89557, USA; E-Mails: suarezf@unr.edu (F.S.); styler@unr.edu (S.W.T.)

[2] Department of Evolutionary Anthropology, Duke University, Campus Box 90383, Durham, NC 27708, USA; E-Mail: glander@duke.edu

[3] Civil Engineering and Geosciences, TU Delft, P.O. Box 5048, 2600 GA, Delft, Netherlands; E-Mail: n.c.vandegiesen@tudelft.nl

[4] Department of Biological and Ecological Engineering, Oregon State University, 116 Gilmore Hall, Corvallis, OR 97331, USA; E-Mail: john.selker@oregonstate.edu

* Author to whom correspondence should be addressed; E-Mail: mhausner@unr.edu; Tel.: +1-775-784-1239; Fax: +1-775-784-1833.

Received: 19 October 2011; in revised form: 15 November 2011 / Accepted: 16 November 2011 / Published: 21 November 2011

Derivation of the Relationships Between Raman Spectra Signals and Temperature

A DTS instrument pulses a laser light at a specific wavelength into an optical fiber, and this light is scattered as it travels down the fiber by interactions between the photons and the crystalline structure of the glass fiber. This scattering takes three forms: Rayleigh, Brillioun, and Raman scattering. Rayleigh scattering is the result of elastic collisions, and the scattered (reflected) signal has the same wavelength as the incident light. Brillioun and Raman spectra scattering are both inelastic events, and result in scattered signals comprising the red-shifted Stokes and the blue-shifted anti-Stokes components. In Brillioun scattering, the amplitudes of the Stokes and anti-Stokes signals are predictable given the amplitude of the incident light, but the wavelengths of the resulting signals are variable [1]. In contrast, the Raman spectra Stokes and anti-Stokes signals have predictable wavelengths, but the relative amplitude of the two signals varies with temperature [2]. While Brillioun-based DTS instruments are commercially available [1], the Raman-based systems are more commonly used in hydrology because of the greater temperature accuracies that can be attained.

The Raman Stokes signal is generated when a photon excites a vibrating molecule at a base excitation state and the molecule returns to a slightly higher vibrational state. When the incident photon hits a faster-vibrating molecule, *i.e.*, one at a higher temperature, and this molecule then returns to a lower state, the anti-Stokes signal is generated. The warmer the optical fiber, the more frequently these previously-excited molecules will be encountered [2]. The anti-Stokes signal is strongly dependent on the temperature of the fiber, while the Stokes signal is only slightly affected by temperature changes. Because of this difference in temperature dependence, the relative strengths of

these signals can be used to calculate the temperature at the point of the scattering. With the known speed of light in the optical fiber, optical time domain reflectometry (OTDR) principles can be used to determine the point on the fiber from which the light was scattered.

Farahani and Gogolla [3] provide an excellent, in-depth discussion of the physics that control Raman spectra scattering in optical fibers, and Rogers [4] and Yilmaz and Karlik [5] present a similar work. Suárez *et al.* [6] present a general discussion of DTS calibration methods focused on a general assessment of DTS installations. This paper presents a more detailed discussion of DTS calibrations, including a number of different methodologies for the user to choose from. Because of some variations in the symbols and terminology used in different publications, we will derive the equations relating the power of the Raman signals to temperature. The power of the anti-Stokes and Stokes signals (P_{AS} and P_S, respectively) scattered from a short section of fiber of length Δz is given by [3],

$$\Delta P_{AS} = \wp_{AS}\Gamma_{AS}P_0 \cdot \Delta z \tag{1}$$

$$\Delta P_S = \wp_S\Gamma_S P_0 \cdot \Delta z \tag{2}$$

where $\wp_{AS}$ and $\wp_S$ are the chance that a particle has certain quantum state according to Bose-Einstein statistics, for the anti-Stokes and Stokes signals, respectively; Γ_{AS} and Γ_S are the capture coefficients (the fraction of the scattered light that is directed back into the fiber towards the initial light source as a function of the number of and types of molecules in the volume determined by Δz) of the anti-Stokes and Stokes signals, respectively; and P_0 is the power of the laser pulse. The chances that a particle has a certain quantum state according to Bose-Einstein statistics of the Stokes and anti-Stokes signals are given by [3],

$$\wp_S = \frac{1}{1-\exp\left(-\Delta E/kT\right)} \tag{3}$$

$$\wp_{AS} = \frac{\exp\left(-\Delta E/kT\right)}{1-\exp\left(-\Delta E/kT\right)} \tag{4}$$

where T is the temperature of the fiber at the point of scattering, ΔE is the frequency shift between the incident light and the scattered Raman signals, and k is the Boltzmann constant. Equations (1) and (2) can be integrated to return functions of $P_{AS}(z)$ and $P_S(z)$, the power of the scattered Raman spectra signals over Δz. Attenuation of the incident light in the fiber and summation of Equations (1) and (2) over z yields, according to Beer's law:

$$P_{AS}(z) = \wp_{AS}\Gamma_{AS}\hat{P}_0\frac{1}{\alpha_0}\exp(-\alpha_0 z) \tag{5}$$

$$P_S(z) = \wp_S\Gamma_S\hat{P}_0\frac{1}{\alpha_0}\exp(-\alpha_0 z) \tag{6}$$

where $\hat{P}_0$ is the power of the light pulse injected into the fiber by the instrument and α_0 (m^{-1}) is the attenuation constant of the incident laser light. The exponential term in Equations (5) and (6) describes the attenuation of the incident light according to Beers' Law as it travels through the fiber. In the equations presented here, the attenuation constant α_0 is also assumed to be uniform along the entire length of the fiber.

After the Raman scattering event, the scattered light travels back from the point of scatter to the instrument's photon detectors at $z = 0$. Over this length of fiber, both the anti-Stokes and Stokes signals are also attenuated according to Beers' Law. Including this attenuation in Equations (5) and (6) yields:

$$P_{AS}(z) = \wp_{AS}\Gamma_{AS}\hat{P}_0 \frac{1}{\alpha_0}\exp(-\alpha_0 z)\exp(-\alpha_{AS} z) \tag{7}$$

$$P_S(z) = \wp_S\Gamma_S\hat{P}_0 \frac{1}{\alpha_0}\exp(-\alpha_0 z)\exp(-\alpha_S z) \tag{8}$$

where α_{AS} and α_S are the attenuation coefficients of the anti-Stokes and Stokes signals, respectively, which are also assumed to be uniform along the fiber. Taking the ratio of Equations (7) and (8) yields:

$$\frac{P_{AS}}{P_S}(z) = \frac{\wp_{AS}}{\wp_S}\frac{\Gamma_{AS}}{\Gamma_S}\frac{\exp(-\alpha_{AS} z)}{\exp(-\alpha_S z)} \tag{9}$$

The ratio $\frac{\Gamma_{AS}}{\Gamma_S}$ can be approximated as $\left(\frac{\lambda_{AS}}{\lambda_S}\right)^4$, where λ_{AS} and λ_S are the wavelengths of the anti-Stokes and Stokes signals, respectively [5]. Making this simplification and substituting the values of the Bose-Einstein probability functions, Equation (9) yields:

$$\frac{P_{AS}}{P_S}(z) = \exp\left(-\frac{\Delta E}{kT}\right)\left(\frac{\lambda_{AS}}{\lambda_S}\right)^4 \exp(-\Delta\alpha z) \tag{10}$$

where $\Delta\alpha = \alpha_{AS} - \alpha_S$. Because the attenuation of the anti-Stokes signal will always be greater than the attenuation of the Stokes signal as it is shorter in wavelength, $\Delta\alpha$ will always be greater than zero. Equation (10) describes the approximated theoretical ratio of the anti-Stokes to Stokes signal present in the fiber at the point where the light was injected and where the scattered Raman spectra signals can be detected ($z = 0$).

To calculate the ratio of anti-Stokes to Stokes power that is actually *detected* by the instrument, Farahani and Gogolla [3] added an additional term $\frac{R_{AS}}{R_S}$ into Equation (10), which quantifies the relative efficiencies of the anti-Stokes and Stokes photon receptors used in the DTS instrument, integrated over the relevant wavelengths. Incorporating this term into Equation (10) and then solving for T yields [3]:

$$T\left(z, \frac{P_S}{P_{AS}}\right) = \frac{\Delta E / k}{\ln\frac{P_S}{P_{AS}} + \ln\frac{R_{AS}}{R_A} + \ln\left[\left(\frac{\lambda_S}{\lambda_{AS}}\right)^4\right] - \Delta\alpha z} \tag{11}$$

In Equation (11), the values of P_{AS} and P_S correspond to the anti-Stokes and Stokes signals observed by the DTS instrument. The values of ΔE, R_S, and R_{AS} depend on the DTS instrument—these terms are controlled by the wavelength and frequency of the incident laser and the shifted Raman signals. While these wavelengths depend primarily on the construction of the instrument, they may also be affected slightly by the operating conditions of the DTS laser, including temperature, humidity, and small fluctuations in the power supply to the unit. The value of $\Delta\alpha$ is affected by these factors, but also by the selection and the physical condition of the optical fiber itself. To simplify the calibration of the raw Raman data collected by the DTS, Equation (11) is re-written to the following form:

$$T(z) = \frac{\gamma}{\ln\frac{P_S(z)}{P_{aS}(z)} + C - \Delta\alpha z} \quad (12)$$

In Equation (12), $\gamma = \frac{\Delta E}{k}$ and the second and third terms in the denominator of Equation (11) have been combined into a single dimensionless calibration parameter *C*. This equation is used as the basis for the calibration routines presented in the paper.

References

1. Selker, J.S.; Thévanaz, L.; Huwald, H.; Mallet, A.; Luxemburg, W.; van de Giesen, N.; Stejskal, M.; Zeman, J.; Westhoff, M.C.; Parlange, M.B. Distributed fiber-optic temperature sensing for hydrologic systems. *Water Resour. Res.* **2006**, *42*, doi:10.1029/2006WR005326.
2. Smith, E.; Dent, G. *Modern Raman Spectroscopy: A Practical Approach*; Wiley: Hoboken, NJ, USA, 2005; pp. 1-22.
3. Farahani, M.A.; Gogolla, T. Spontaneous Raman scattering in optical fibers with modulated probe light for distributed temperature Raman remote sensing. *J. Lightwave Tech.* **1999**, *17*, 1379-1391.
4. Rogers, A. Review article: Distributed optical-fibre sensing. *Meas. Sci. Tech.* **1999**, *10*, R75-R99.
5. Yilmaz, G.; Karlik, S.E. A distributed optical fiber sensor for temperature detection in power cables. *Sens. Actuat. A* **2006**, *125*, 148-155.
6. Suárez, F.; Aravena, J.E.; Hausner, M.B.; Childress, A.E.; Tyler, S.W. Assessment of a vertical high-resolution distributed-temperature-sensing system in a shallow thermohaline environment. *Hydrol. Earth Syst. Sci.* **2011**, *15*, 1081-1093.

© 2011 by the authors; licensee MDPI, Basel, Switzerland. This article is an open access article distributed under the terms and conditions of the Creative Commons Attribution license (http://creativecommons.org/licenses/by/3.0/).
